# Plasma markers of oxidative stress are uncorrelated in a wild mammal

**DOI:** 10.1002/ece3.1771

**Published:** 2015-10-19

**Authors:** Louise L. Christensen, Colin Selman, Jonathan D. Blount, Jill G. Pilkington, Kathryn A. Watt, Josephine M. Pemberton, Jane M. Reid, Daniel H. Nussey

**Affiliations:** ^1^ Institute of Biological and Environmental SciencesUniversity of AberdeenAberdeenUK; ^2^ Glasgow Ageing Research Network (GARNER)Institute of BiodiversityAnimal Health and Comparative MedicineUniversity of GlasgowGlasgowUK; ^3^ College of Life and Environmental SciencesUniversity of ExeterPenryn CampusUK; ^4^ Institute of Evolutionary BiologyUniversity of EdinburghEdinburghUK

**Keywords:** Antioxidants, life history, oxidative damage, plasma, reactive oxygen species, redox, Soay sheep

## Abstract

Oxidative stress, which results from an imbalance between the production of potentially damaging reactive oxygen species versus antioxidant defenses and repair mechanisms, has been proposed as an important mediator of life‐history trade‐offs. A plethora of biomarkers associated with oxidative stress exist, but few ecological studies have examined the relationships among different markers in organisms experiencing natural conditions or tested whether those relationships are stable across different environments and demographic groups. It is therefore not clear to what extent studies of different markers can be compared, or whether studies that focus on a single marker can draw general conclusions regarding oxidative stress. We measured widely used markers of oxidative damage (protein carbonyls and malondialdehyde) and antioxidant defense (superoxide dismutase and total antioxidant capacity) from 706 plasma samples collected over a 4‐year period in a wild population of Soay sheep on St Kilda. We quantified the correlation structure among these four markers across the entire sample set and also within separate years, age groups (lambs and adults), and sexes. We found some moderately strong correlations between some pairs of markers when data from all 4 years were pooled. However, these correlations were caused by considerable among‐year variation in mean marker values; correlation coefficients were small and not significantly different from zero after accounting for among‐year variation. Furthermore, within each year, age, and sex subgroup, the pairwise correlation coefficients among the four markers were weak, nonsignificant, and distributed around zero. In addition, principal component analysis confirmed that the four markers represented four independent axes of variation. Our results suggest that plasma markers of oxidative stress may vary dramatically among years, presumably due to environmental conditions, and that this variation can induce population‐level correlations among markers even in the absence of any correlations within contemporaneous subgroups. The absence of any consistent correlations within years or demographic subgroups implies that care must be taken when generalizing from observed relationships with oxidative stress markers, as each marker may reflect different and potentially uncoupled biochemical processes.

## Introduction

The balance between production of potentially damaging reactive oxygen species (ROS) and the mobilization of antioxidant (AOX) defenses and repair systems that prevent damage is of fundamental importance to cellular and organismal function (Balaban et al. 2005; Monaghan et al. 2009). ROS are primarily produced as by‐products of oxidation–reduction (redox) reactions (Beckman and Ames 1998; Dowling and Simmons 2009), which occur during aerobic metabolism within the mitochondria. They are important components of cellular signaling (e.g., Nemoto et al. 2000) and immune function (Babior et al. 1973; Forman and Torres 2002), but in excess can cause DNA, lipid, and protein damage and disrupt cellular function (Harman 1956; Beckman and Ames 1998; Buffenstein et al. 2008). A suite of dietary and endogenous antioxidants (AOX) exists to quench ROS and protect key biomolecules (Finkel and Holbrook 2000). Oxidative stress (OS) has been defined as “an imbalance between oxidants and AOX in favor of the oxidants” (Sies and Jones 2007). This arises when ROS production exceeds the capacity of AOX defenses, potentially leading to disruption of redox signaling and cellular damage and dysfunction (Sies 1991; Sies and Jones 2007; Limón‐Pacheco and Gonsebatt 2009).

Oxidative stress consists of four components: ROS production, AOX defense, oxidative damage, and oxidative repair (Beckman and Ames 1998). Due to the short half‐life of ROS, measuring production in vivo is extremely challenging (Halliwell 1991; De Lamirande et al. 1997; Rimbach et al. 1999; Finaud et al. 2006), and measuring the activity of repair systems in vivo is also difficult (Monaghan et al. 2009). Whole organism biologists have therefore understandably focused on indirect biomarkers of OS, such as damage to proteins, lipids, and DNA. Measurement of different AOX molecule levels, or assays testing the cellular capacity to cope with an oxidative challenge, is also commonly applied (Monaghan et al. 2009).

To adequately quantitate the overall redox state of an organism, it has been suggested that researchers should to apply at least one marker of AOX protection as well as at least one marker of oxidative damage (Prior and Cao 1999; Clarkson and Thompson 2000; Cohen and McGraw 2009; Costantini and Verhulst 2009; Selman et al. 2012). However, any attempt to generalize findings across studies using different AOX and damage markers rests on an assumption that different markers reflect a unified redox system at the organismal level, and thus, that ROS will induce similar levels of damage across DNA, lipids, and proteins, as well as similar responses across AOX systems. Thus far, the choice of biomarkers has varied immensely across studies and species; more than a hundred methods can be used to measure OS (Dotan et al. 2004: for reviews see, e.g., Dalle‐Donne et al. 2006; Monaghan et al. 2009). However, the degree to which different markers are, in fact, correlated across individuals within any study, and hence to which any single marker can be interpreted to measure some aspect of a unified redox system, has rarely been examined.

OS is hypothesized to be an important mediator of life‐history trade‐offs (Stearns 1992; Beckman and Ames 1998; Monaghan et al. 2009; Metcalfe and Alonso‐Alvarez 2010; Selman et al. 2012). In the context of life‐history theory, investment in metabolically demanding activities such as growth and reproduction (Ellison 2003; Wiersma et al. 2004; Arnott et al. 2006; Criscuolo et al. 2008), which are essential components of fitness, is predicted to produce ROS and also draws limited resources away from AOX defense and repair systems. This is hypothesized to induce OS and, ultimately, lead to an accumulation of damage, reduced maintenance function, and organismal senescence (Harman 1956; Beckman and Ames 1998; Isaksson et al. 2011; Selman et al. 2012). Studies in both experimental and wild populations have demonstrated associations between markers of OS and life‐history traits (e.g., Alonso‐Alvarez et al. 2007; Bize et al. 2008; Bergeron et al. 2011; Losdat et al. 2011; Saino et al. 2011; Wilson et al. 2012; Marri and Richner 2014). Laboratory studies that manipulate oxidative challenge or life‐history investment are pivotal to our ability to establish whether OS may underpin life‐history variation (Metcalfe and Monaghan 2013), and there is emerging experimental evidence that supports this overarching hypothesis (Sohal et al. 1994; Wiersma et al. 2004; Bertrand et al. 2006;. However, see also Selman et al. 2008; who found no significant effects of lifelong cold exposure on mortality, oxidative damage, or AOX protection in captive short‐tailed field voles (*Microtus agrestis*); Ołdakowski et al. 2012; who found no effect of reproduction on oxidative damage to heart, kidney, or skeletal muscle in captive bank voles (*Myodes glareolus*); and Brzęk et al. 2014; who found no effect of reproduction on oxidative damage to lipids in two lines of laboratory mice (*Mus musculus*), selected either for high basal metabolic rate (H‐BMR) or low basal metabolic rate (L‐BMR)). Complementary studies of the relationships between OS, life‐history traits, and fitness from the wild are also necessary if we wish to understand how natural selection and environmental variation actually shape the redox balance and its fitness costs and benefits under ecologically realistic conditions (Selman et al. 2012). However, both types of studies have typically only used one or a few markers of OS, with little consistency across studies in the choice of markers (Monaghan et al. 2009).

In order to understand the degree to which different studies might be measuring similar biological effects, we need to understand the strength and consistency of correlations among different markers of OS. While one might predict that strong positive correlations should exist between different oxidative damage markers, the direction of correlations among AOX markers and their relationships with damage markers are less easy to predict. AOX markers may reflect the same overall axis of variation and hence be positively correlated or may compensate for one another's availability and thus be negatively correlated (Cohen and McGraw 2009). Similarly, while organisms with impaired AOX defenses might sustain high levels of oxidative damage, AOX function could, in fact, be upregulated in response to damage (Sepp et al. 2012; Alan and McWilliams 2013). However, regardless of the direction of correlations, if a particular set of OS markers were indeed reflecting a unified redox system of molecular damage and AOX defense at the organismal level, meaning that single markers could be interpreted to capture general redox variation, we would expect strong, highly significant correlations among markers that remain broadly stable across environmental conditions, ages, and sexes. Few studies to date have quantified the correlations among different OS biomarkers in either natural or experimental populations of nonmodel organisms of interest in evolutionary ecology. Some avian studies report evidence of correlations among OS markers (e.g., in captive zebra finches (*Taeniopygia guttata*), Costantini et al. 2011), whereas others did not detect any such correlations (e.g., in wild‐caught captive greenfinches (*Carduelis chloris),* Sepp et al. 2012). Even fewer studies have quantified the degrees to which such correlations remain consistent with respect to environment, age, or sex (although see, e.g., Costantini et al. 2013; Romero‐Haro and Alonso‐Alvarez 2014, which respectively quantitate variation with flight activity and age in zebra finches).

Here, we quantitate the phenotypic correlations among two commonly used markers of oxidative damage, protein carbonyls (PC) and malondialdehyde (MDA), and two commonly used AOX markers, superoxide dismutase (SOD) and total antioxidant capacity (TAC). We used plasma samples collected from a Soay sheep (*Ovis aries*) population experiencing variable natural environmental conditions. Our samples were collected over 4 years, during which population size and climate conditions varied considerably and samples were collected from animals from different age and sex groups. We tested whether correlations observed across the entire sample set were driven by among‐year or among‐group variation in our OS markers, or driven by consistent associations within years and groups. We thereby quantitate the degree to which these four markers can be interpreted as interchangeable measures of an overall redox system.

## Materials and Methods

### Study system & sample collection

We studied an unmanaged and unpredated population of Soay sheep resident in the Village Bay area of the island of Hirta in the St. Kilda archipelago (57°49′N 8°34′W). Since 1985, this population has been monitored intensively: individuals are uniquely tagged at birth and followed throughout their lifetime (Clutton‐Brock & Pemberton, 2004). Each year, in August, as many resident sheep as possible are rounded up and caught in temporary corral traps. Upon capture, a variety of biometric measures are taken, along with a blood sample. Blood is collected into 9‐mL lithium heparin vacutainers by venepuncture. For this study, we used blood samples collected during each August in 2010–2013 inclusive from animals of known age and sex. Following collection, whole blood was stored at 4°C until it was processed the following day. Whole blood was spun at 1008 g for 10 min and the plasma supernatant was collected and stored at −80°C until further use. Samples were transported from St Kilda to our laboratories using either liquid nitrogen vapor shippers (2010 and 2011) or a portable −80°C freezer (2012 and 2013: Stirling Shuttle Ultra Low Portable Freezer. Triple Red Laboratory Technology, Bucks, England).

### Laboratory methods

The four biomarkers were assayed as follows. To quantitate PC, plasma protein concentration (mg/mL) was determined using the Bradford method (Bradford, 1976). Absorbance was measured at 595 nm on a Spectra Max plate reader (Molecular Devices, Sunnyvale, CA, USA).

Intraplate repeatability of this nonkit assay was 0.92 across 22 samples run in triplicate (Appendix S1). Samples were diluted to 5 mg/mL protein content and stored at −80°C until further use. Plasma PC content (nmol/mg protein) was determined using a Cayman Protein Carbonyl Assay Kit (ID: 10005020; Cayman Chemical Company, Ann Arbour, Michigan, USA). 2,4‐dinitrophenylhydrazine (DNPH) was added to initiate a reaction with PCs leading to the formation of a hydrazone detectable by spectrophotometry. The amount of PCs was standardized to protein concentration by dividing by protein content. We followed the manufacturer's protocol, with one exception: when pellets were washed and resuspended 3× with (1:1) ethanol/acetate, we added 500 *μ*L ethanol/acetate per sample per wash, rather than 1 mL, as specified. Absorbance was measured at 370 nm on a plate reader (Spectra Max Molecular Devices, Sunnyvale, CA, USA). Data were analyzed using Softmax Pro 5.3 software. The manufacturer's stated interplate coefficient of variation (CV) for this assay is 8.5%.

High‐performance liquid chromatography (HPLC) was used to quantitate MDA levels (*μ*M/L) of plasma, following Nussey et al. (2009). The HPLC method is considered more accurate than ultraviolet visible spectrophotometry (UV–vis), because the UV–vis method lacks specificity and can be affected by other UV–vis species (Lovrić, et al., 2008), but the HPLC method is specific to MDA (Lopez‐Torres et al., 1993). Samples were vortexed and centrifuged before use to ensure sufficient mixing and to avoid any debris. About 50 *μ*L butylated hydroxytoluene solution, 400 *μ*L 0.44 M phosphoric acid solution, and 100 *μ*L (42 mm) thiobarbituric acid (TBA) solution were added to 50 *μ*L sample or standard (1,1,3,3‐tetraethoxypropane, TEP). The samples were vortexed for 5 sec and heated to 100°C for 1 h in a dry bath incubator. Samples were cooled for 5 min on ice, spun down, and 200 *μ*L *n*‐butanol was added to each tube followed by vortexing for 20 s. Tubes were centrifuged at 15,338 g for 3 min at 4°C, and 90 *μ*L of the upper phase was collected and stored in a HPLC vial for analysis. About 40 *μ*L of sample was injected into a HPLC system (Dionex Corporation, Sunnyvale, CA, USA) fitted with a 5 *μ*m ODS guard column and a Hewlett‐Packard Hypersil 5 *μ* ODS 100 × 4.6 mm column oven maintained at 37°C. Methanol buffer (40:60, 50 mm anhydrous solution of potassium monobasic phosphate, pH 6.8) was running isocratically over 3.75 min (1 mL/min) as the mobile phase. A fluorescence detector (RF2000; Dionex) set at 515 nm (excitation) and 553 nm (emission) recorded the data. A standard curve was prepared from a TEP stock solution, following serial dilution with 40% ethanol and this was used for calibration. We estimated the interplate repeatability of this nonkit assay at 0.91 from 58 repeat samples (Appendix S2).

Plasma total SOD activity (U/mL) was determined using Cayman's Superoxide Dismutase Assay Kit (ID: 706002; Cayman Chemical Company) following the manufacturer's protocol. SOD activity was estimated by measuring the dismutation of superoxide radicals and calibrating this against a bovine erythrocyte SOD (Cu/Zn) enzyme standard curve. Absorbance was read at 450 nm, and data were analyzed using Softmax Pro 5.3 software. 2010 and 2011 samples were analyzed using a Molecular Devices SpectraMax M2 plate reader, and 2012 and 2013 samples were analyzed using a SpectraMax plate reader. The manufacturer's stated interplate CV is 3.7%.

Plasma TAC levels (mm) were estimated using Cayman's Antioxidant Assay Kit (ID: 709001; Cayman Chemical Company) following the manufacturer's protocol. A Trolox standard curve was used to quantitate the antioxidant capacity of the sample, measured in millimolar Trolox equivalents. 2010 and 2011 samples were analyzed using a Molecular Devices SpectraMax M2 plate reader, 2012 samples were analyzed using a SpectraMax plate reader, and 2013 samples were analyzed on a Thermo Scientific Multiscan GO plate reader. Absorbance was read at either 405 or 750 nm. The manufacturer's stated interplate CV is 3%.

### Statistical analysis

We initially calculated Pearson product‐moment correlation coefficients (r) between all pairwise combinations of the four markers using all available data. We then grouped our samples by year, sex, and two different age classes: lambs (<1 year old) and adults (>1 year old). Since few 1‐year‐old animals were caught over the 4 years of our study (primarily due to very high lamb mortality), and previous research suggests demographic differences among yearlings and other age groups (Coulson et al. 2001), we excluded the few sampled yearlings from further analyses. Sample sizes for each year, sex, and age group are presented in Table 1. We calculated the means and standard deviations of each marker in each year, age, and sex group and in all possible subgrouping combinations within these. This created a total of 16 subgroups – female and male lambs and adults within each of the 4 years. These analyses revealed substantial among‐year variation in the means of some of the markers (see Results). We therefore tested whether overall correlations between markers across all sampled individuals were caused solely by among‐year variation in means by z‐score‐standardizing marker values within years and quantitating correlations among these standardized values. *z*‐scores were obtained by subtracting the population means from each observation and dividing by the population standard deviation. While the distribution of the raw marker data deviated somewhat from normality, the z‐score‐corrected data were approximately normally distributed. We then quantified correlations within each year, age, and sex grouping and within all 16 subgroups where the sample size exceeded 15 (one group, adult males in 2010 was not analyzed separately due to the small sample size, Table 1). We applied a sequential Holm–Bonferroni correction across all tests to adjust *P*‐values for multiple testing (Holm, 1979) and minimize the risk of type I errors. The analyzed data subgroups of adult sheep pooled across all 4 years contained repeat observations of some individuals (one individual was sampled in all 4 years, 55 individuals were sampled in 3 years, and 89 individuals were sampled in 2 years). Therefore, to account for correlations in marker values within individuals in the context of hypothesis testing, we fitted mixed models with random individual effects. Although these models estimate the slope of the relationship between one marker and another rather than the correlation, their results did not differ from those drawn from the correlation analysis. As our primary aim was to quantitate the correlations among markers, we solely ‐present the correlations for simplicity.

**Table 1:** Number of individual Soay sheep for which oxidative stress markers were measures, stratified by year of collection, age group, and sex

	2010		2011		2012		2013		Total	
	Lamb	Adult	Lamb	Adult	Lamb	Adult	Lamb	Adult	Lamb	Adult
Female	26	34	49	118	30	80	60	81	165	313
Male	20	5	50	43	21	17	56	16	147	81
Total	46	39	99	161	51	97	116	97	312	394

We additionally used principal component analysis (PCA) to assess whether oxidative status of individuals, as assessed by our four focal markers, can be described by fewer than four independent axes, implying that individual markers could be interpreted as measures of a unified redox system. PCA was applied to PC, MDA, SOD, and TAC data (raw values) for all years combined and for each year separately to obtain loadings for each principal component axis. Data were z‐score‐transformed in order to estimate the proportion of variance and cumulative variation explained by each axis on a comparable scale. Statistical analyses were performed using the statistical software program R version 2.15.2 (R Core Team 2012).

## Results

**Figure 1:**
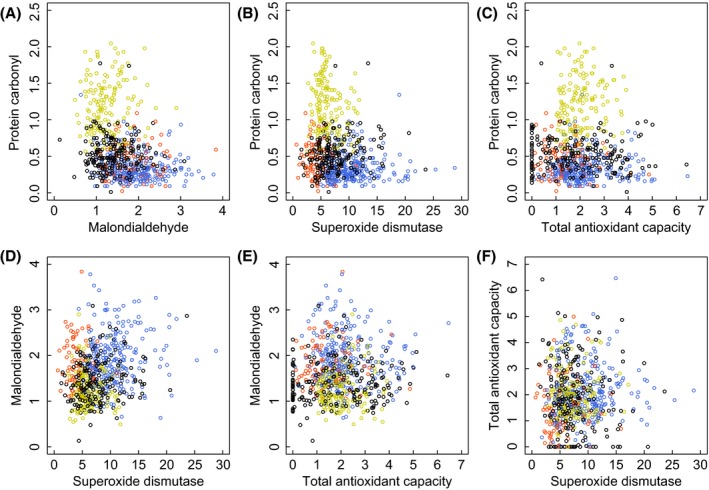
Correlations among four markers of oxidative stress. (A) protein carbonyl (“PC,” nmol/mg protein) and malondialdehyde (“MDA,” *μ*M/L), (B) PC and superoxide dismutase (“SOD,” U/mL), (C) PC and total antioxidant capacity (“TAC,” mm), (D) MDA and SOD, (E) MDA and TAC, and (F) TAC and SOD. Orange = 2010, blue = 2011, green = 2012, and black = 2013 data.

We detected moderately strong and statistically significant correlations between some markers when data from all individuals sampled in all 4 years were pooled (Fig. 1, Table 2). PC and MDA were significantly negatively correlated (*r* = −0.34), while SOD was negatively correlated with PC (*r* = −0.24) but positively correlated with MDA (*r* = 0.25; Table 2). The negative PC–MDA correlation was similarly strong and significant within all age and sex group combinations, although the correlations with SOD were less consistent (Table 1). All other correlations across years were weak and nonsignificant except for SOD–TAC which was marginally significantly positive within the adult group (r = 0.18) and within adult females but not within adult males (Table 2).

**Table 2:** Pearson product‐moment correlation coefficients of untransformed data (*r*), of *z*‐score‐transformed data (*z* (*r*)) and Holm–Bonferroni adjusted *P*‐values for the correlations between different markers of oxidative stress; protein carbonyl (PC), malondialdehyde (MDA), superoxide dismutase (SOD), and total antioxidant capacity (TAC). Samples were split by sex, by age, and by both sex and age. Data for adult males in 2010 were not analyzed as a separate group due to small sample size (*n* = 5)

Marker/s	All years			2010		2011		2012		2013	
	*r*	*P*	*z* (*r*)	*r*	*P*	*r*	*P*	*r*	*P*	*r*	*P*
All animals											
PC‐MDA	−0.34	<0.001***	−0.01	0.05	1.00	−0.08	1.00	0.07	1.00	−0.01	1.00
PC‐SOD	−0.24	<0.001***	0.02	−0.18	1.00	0.07	1.00	−0.21	0.29	0.22	**0.06**
PC‐TAC	<0.01	1.00	0.02	−0.11	1.00	0.07	1.00	0.11	1.00	−0.05	1.00
MDA‐SOD	0.25	<0.001***	0.09	−0.06	1.00	0.12	1.00	−0.03	1.00	0.21	**0.09**
MDA‐TAC	0.05	1.00	0.05	0.03	1.00	0.01	1.00	0.09	1.00	0.08	1.00
SOD‐TAC	0.06	1.00	−0.06	0.10	1.00	0.02	1.00	−0.28	0.02*	−0.07	1.00
All Lambs											
PC‐MDA	−0.31	<0.001***	−0.05	0.17	1.00	−0.14	1.00	−0.01	1.00	−0.09	1.00
PC‐SOD	−0.06	1.00	0.03	−0.21	1.00	0.02	1.00	−0.17	1.00	0.23	0.56
PC‐TAC	−0.01	1.00	0.04	0.20	1.00	0.01	1.00	0.27	1.00	−0.10	1.00
MDA‐SOD	0.18	**0.06**	0.11	−0.06	1.00	0.11	1.00	0.03	1.00	0.23	0.56
MDA‐TAC	0.02	1.00	0.04	0.09	1.00	0.01	1.00	<−0.01	1.00	0.06	1.00
SOD‐TAC	0.01	1.00	0.04	−0.10	1.00	0.18	1.00	0.08	1.00	−0.05	1.00
♀ Lambs											
PC‐MDA	−0.29	0.009**	0.01	0.23	1.00	−0.03	1.00	0.09	1.00	−0.11	1.00
PC‐SOD	−0.04	1.00	0.04	−0.15	1.00	−0.18	1.00	−0.09	1.00	0.41	**0.06**
PC‐TAC	−0.01	1.00	0.09	0.42	1.00	−0.04	1.00	0.34	1.00	−0.08	1.00
MDA‐SOD	0.27	0.02*	0.14	0.04	1.00	0.22	1.00	−0.12	1.00	0.26	1.00
MDA‐TAC	−0.04	1.00	−0.04	0.04	1.00	0.06	1.00	−0.24	1.00	−0.06	1.00
SOD‐TAC	−0.09	1.00	0.01	−0.19	1.00	0.19	1.00	0.21	1.00	−0.17	1.00
♂ Lambs											
PC‐MDA	−0.34	<0.001***	−0.13	0.12	1.00	−0.29	1.00	−0.10	1.00	−0.08	1.00
PC‐SOD	−0.07	1.00	0.04	−0.27	1.00	0.20	1.00	−0.27	1.00	0.11	1.00
PC‐TAC	−0.01	1.00	−0.01	−0.10	1.00	0.07	1.00	0.17	1.00	−0.11	1.00
MDA‐SOD	0.06	1.00	0.05	−0.27	1.00	−0.02	1.00	0.18	1.00	0.18	1.00
MDA‐TAC	0.11	1.00	0.17	0.23	1.00	−0.01	1.00	0.21	1.00	0.28	1.00
SOD‐TAC	0.13	1.00	0.10	0.07	1.00	0.18	1.00	−0.13	1.00	0.13	1.00
All Adults											
PC‐MDA	−0.36	<0.001***	0.03	–	–	−0.04	1.00	0.11	1.00	0.12	1.00
PC‐SOD	−0.37	<0.001***	0.06	–	–	0.10	1.00	0.01	1.00	0.16	1.00
PC‐TAC	−0.02	1.00	0.03	–	–	0.15	1.00	−0.10	1.00	0.06	1.00
MDA‐SOD	0.32	<0.001***	0.08	–	–	0.18	0.56	−0.11	1.00	0.16	1.00
MDA‐TAC	0.07	1.00	0.04	–	–	−0.03	1.00	0.13	1.00	0.10	1.00
SOD‐TAC	0.18	0.02*	0.03	–	–	0.11	1.00	−0.13	1.00	−0.10	1.00
♀ Adults											
PC‐MDA	−0.34	<0.001***	0.05	−0.01	1.00	−0.02	1.00	0.13	1.00	0.09	1.00
PC‐SOD	−0.40	<0.001***	0.03	−0.25	1.00	0.08	1.00	0.01	1.00	0.08	1.00
PC‐TAC	−0.05	1.00	−0.04	−0.27	1.00	0.04	1.00	−0.12	1.00	0.02	1.00
MDA‐SOD	0.34	<0.001***	0.10	−0.05	1.00	0.18	1.00	−0.08	1.00	0.23	1.00
MDA‐TAC	0.10	1.00	0.05	−0.04	1.00	−0.06	1.00	0.16	1.00	0.15	1.00
SOD‐TAC	0.19	0.03*	<−0.01	0.37	0.93	0.11	1.00	−0.18	1.00	−0.15	1.00
♂ Adults											
PC‐MDA	−0.40	0.01*	0.02	–	–	−0.10	1.00	0.02	1.00	0.34	1.00
PC‐SOD	−0.37	0.04*	0.04	–	–	−0.08	1.00	−0.07	1.00	0.48	1.00
PC‐TAC	0.11	1.00	0.28	–	–	0.39	0.35	−0.01	1.00	0.28	1.00
MDA‐SOD	0.39	0.02*	0.12	–	–	0.20	1.00	−0.22	1.00	0.29	1.00
MDA‐TAC	0.02	1.00	−0.01	–	–	0.03	1.00	<−0.01	1.00	−0.12	1.00
SOD‐TAC	0.09	1.00	0.07	–	–	−0.02	1.00	0.18	1.00	0.24	1.00

Bold =  approaching significance at p<0.1, bold and * =  significant at p<0.05; bold and ** =  significant at p<0.01; bold and *** =  significant at p<0.001.

**Figure 2:**
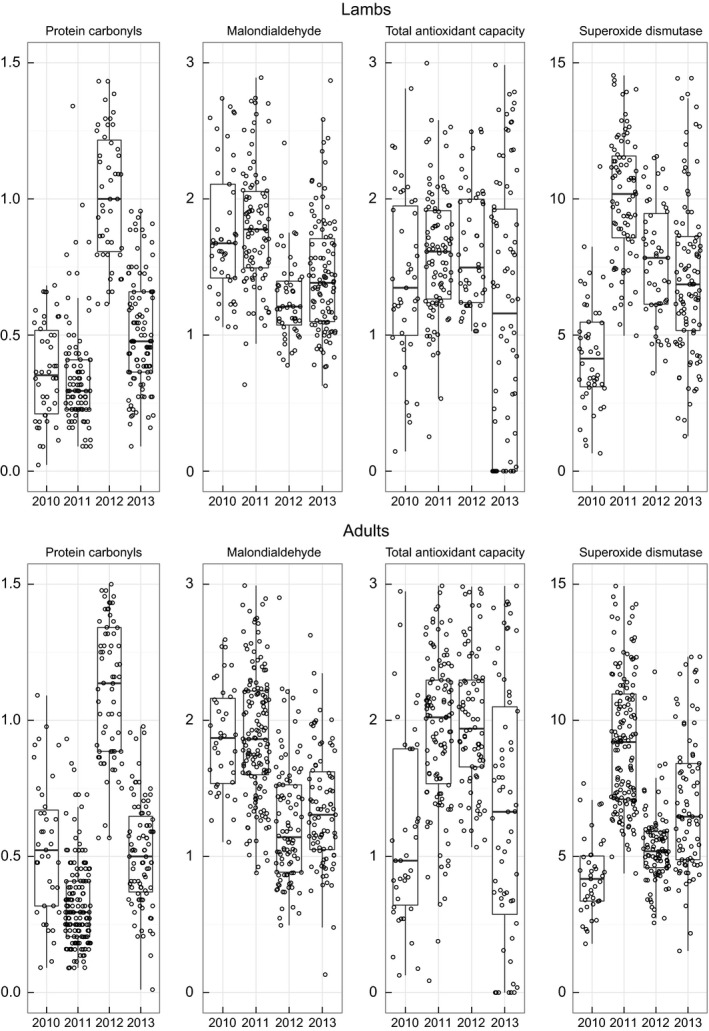
Among‐year variation in protein carbonyls, malondialdehyde, total antioxidant capacity, and superoxide dismutase for lambs (top) and adults (bottom). Median and interquartile range (bar and box); whiskers: 10% and 90% quantiles.

**Figure 3:**
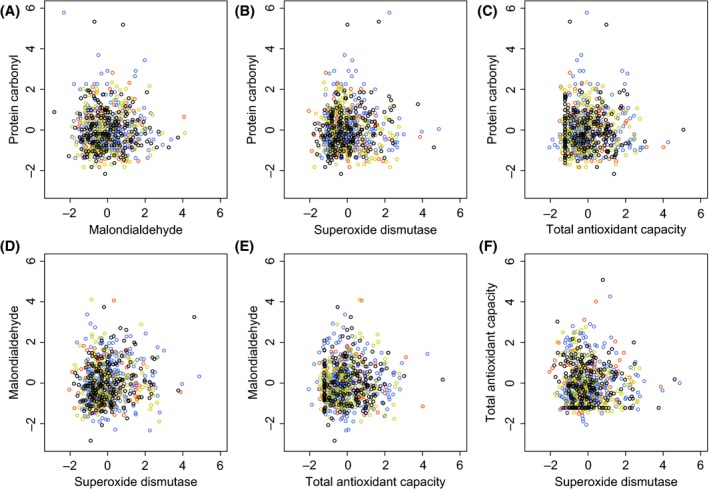
Correlations among four markers of oxidative stress, using *z*‐score‐transformed data. (A) protein carbonyl (“PC,” nmol/mg protein) and malondialdehyde (“MDA,” *μ*M/L), (B) PC and superoxide dismutase (“SOD,” U/mL), (C) PC and total antioxidant capacity (“TAC,” mm), (D) MDA and SOD, (E) MDA and TAC, and (F) TAC and SOD. Orange = 2010, blue = 2011, green = 2012, and black = 2013 data.

However, there was substantial among‐year variation in all four markers, with 2012 in particular showing relatively high mean PC, but low MDA and low SOD (Fig. 2, online Appendix S3). When we *z*‐score transformed the marker data within each year, the correlations between PC and MDA and between PC and SOD became small and did not differ significantly from zero despite the large total sample sizes (Table 2, Fig. 3). This indicates that the moderately strong and significant correlations observed when all 4 years were pooled together were driven by among‐year variation in average marker concentrations and did not reflect covariation in marker values across individual animals within years.

**Figure 4:**
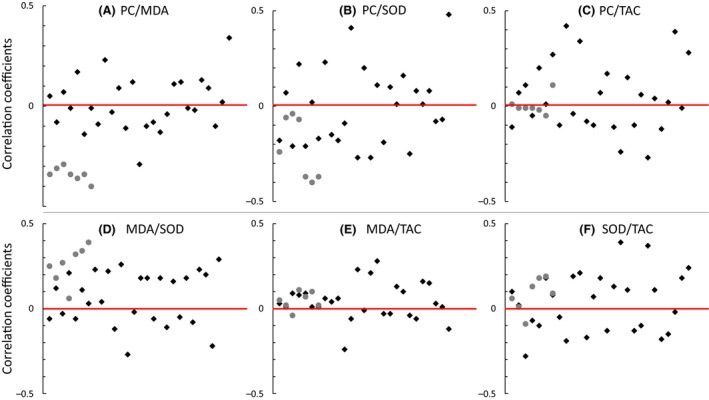
Correlation coefficients among four markers of oxidative stress and their distribution around zero (= red line). (A) protein carbonyl (“PC,” nmol/mg protein) and malondialdehyde (“MDA,” *μ*M/L), (B) PC and superoxide dismutase (“SOD,” U/mL), (C) PC and total antioxidant capacity (“TAC,” mm), (D) MDA and SOD, (E) MDA and TAC, and (F) TAC and SOD. Correlations within individual years and groups = ♦, and correlations across all years pooled = 
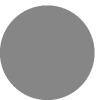
. Random ordering of correlation coefficients along the *x*‐axis.

Correlations among raw values of the four markers within years, sexes, and age groups were generally weak, inconsistent in direction and clustered around zero (Fig. 4; Table 2). Prior to correction for multiple testing, 16 of 156 correlations were significant at the *P* < 0.05 level, but only a single correlation remained nominally significant after Holm–Bonferroni correction (SOD‐TAC, *r* = −0.28, *P* = 0.02. Table 2). Overall, there was little evidence for strong or consistent correlations of any kind among the four OS makers within years, age, or sex groups.

Principal component analysis (PCA) provided further evidence for a lack of covariance among the four OS markers. Specifically, the four individual markers loaded very strongly onto four different principal component axes (Table 3B), and the four PCAs explained similar proportions of the total variance in the z‐score‐standardized marker values (Table 3A). Each of the four principal components, whether estimated across all years or within a single year, explained 16–39% of the overall variance.

**Table 3:** (A) The standard deviation, proportion of variance, cumulative proportion of variance (*z*‐score‐transformed), and (B) marker loadings (raw values) for the principal component analysis (PCA) of our four markers of oxidative stress; proteins carbonyls (PC), malondialdehyde (MDA), superoxide dismutase (SOD), and total antioxidant capacity (TAC)

(A)																				
Importance of components	Principal component 1					Principal component 2					Principal component 3					Principal component 4				
	All years	2010	2011	2012	2013	All years	2010	2011	2012	2013	All years	2010	2011	2012	2013	All years	2010	2011	2012	2013
Standard deviation	1.25	1.13	1.06	1.20	1.14	1.00	1.01	1.05	0.99	1.06	0.89	0.94	0.98	0.95	0.95	0.81	0.91	0.90	0.83	0.84
Proportion of variance	0.39	0.32	0.28	0.36	0.33	0.25	0.26	0.28	0.25	0.28	0.20	0.22	0.24	0.23	0.22	0.16	0.21	0.20	0.17	0.18
Cumulative proportion	0.39	0.32	0.28	0.36	0.33	0.64	0.58	0.56	0.60	0.60	0.84	0.80	0.80	0.83	0.83	1.00	1.00	1.00	1.00	1.00

(B)																				
Marker/s (loadings)	Principal component 1					Principal component 2					Principal component 3					Principal component 4				
	All years	2010	2011	2012	2013	All years	2010	2011	2012	2013	All years	2010	2011	2012	2013	All years	2010	2011	2012	2013
PC	0.03	−0.02	<−0.01	0.04	−0.01	−0.01	−0.03	0.01	−0.04	0.01	0.40	0.03	0.03	0.19	0.03	−0.92	<−0.99	>0.99	0.98	>0.99
MDA	0.04	−0.02	−0.02	0.01	−0.03	−0.03	0.03	<0.01	−0.08	−0.04	−0.92	>0.99	>0.99	0.98	<−0.99	−0.38	0.03	−0.03	−0.20	0.03
SOD	>0.99	>0.99	<−0.99	−0.99	<−0.99	0.02	−0.06	−0.01	−0.10	−0.03	0.04	0.02	<−0.01	0.01	0.03	−0.01	−0.02	<0.01	0.03	−0.02
TAC	0.02	0.06	<−0.01	0.10	0.03	<−0.99	>0.99	>0.99	−0.99	<−0.99	0.02	−0.03	<0.01	−0.08	0.04	0.02	−0.03	0.01	−0.02	0.01

## Discussion

A growing number of studies provide evidence for associations among markers of OS and life‐history traits (Salmon et al. 2001; Alonso‐Alvarez et al. 2007; Catoni et al. 2008; Cohen et al. 2009; Nussey et al. 2009; Archer et al. 2012; Mondy et al. 2012; Rubolini et al. 2012). However, an important question still remains: Can we draw general conclusions about patterns of variation in OS and their consequences for life histories from just one or a few markers, or Are the findings of any one study of OS likely to be specific to the markers selected and the context in which they are measured? Our findings support the latter conclusion. Considering all data pooled across years, we found unexpected negative correlations between our two oxidative damage markers (PC and MDA) which in turn had opposing correlations with one (SOD) but not the other (TAC) of our antioxidant markers. However, we showed that these associations were driven by striking among‐year variation in the means of these four markers, rather than by any underlying or consistent associations between these markers across individual animals sampled within years. Indeed, at the within‐year level, we found no evidence for strong or consistent correlations among any of our markers. Furthermore, principal components analysis supported the conclusion that our four markers capture independent axes of variation in OS phenotype space, and hence, that any one marker provides virtually no information regarding variation in the other markers.

We found that moderate overall correlations among markers estimated across all 4 years combined were caused solely by the substantial among‐year variation in all four markers of OS: All correlations among markers observed across the entire data set became negligible when we accounted for among‐year variation. This suggests that ostensibly linked OS markers can respond very differently to what we assume to be environmental differences among years. For instance, the negative correlation between our two oxidative damage markers, MDA and PC, appeared largely driven by very high PC and low MDA in 2012 relative to other years. The Soay sheep on Hirta follow unstable population dynamics, characterized by years of low and rising numbers followed by high mortality “crash” winters during which over 60% of the population can perish (Clutton‐Brock & Pemberton 2004). Thus, competition for food varies dramatically among years on Hirta, alongside variation in climatic conditions and parasite exposure (Coulson et al. 2001; Hayward et al. 2010). The winter of 2011/2012 was a “crash,” and so, the samples collected in summer 2012 came from animals that had survived a harsh winter and were currently experiencing relatively low competition for food. Why this should have induced an increase in a marker of protein damage (PC) but not in lipid damage (MDA) is unclear. However, it raises the possibility that, under naturally varying conditions, OS markers may be highly plastic and that different markers may respond to environmental variation in contrasting ways. Although it is impossible to control for such variation in wild population studies, several laboratory studies have addressed this by experimentally raising the metabolic investment of individuals, while measuring OS (for some examples, see Alonso‐Alvarez et al. 2004; Ferguson et al. 2008; Lalouette et al. 2011).

Across existing studies, there is no consistent evidence of covariation in different OS markers. A comprehensive meta‐analysis of studies where at least two methods of OS measurement had been applied found that correlations between markers of lipid peroxidation and protein oxidation ranged widely from strong to zero (Dotan et al. 2004). An absence of association could result from the chemical differences in formation of the damage products being measured (Dotan et al. 2004), or due to differences in the rate of repair/or removal of damaged protein versus lipid molecules (Nyström 2005). Previous studies have also observed a lack of consistency in correlations among AOX markers, which again may suggest differences in the biology underlying the formation of different AOX molecules (e.g., Peng et al. 2000; Vider et al. 2001; Dotan et al. 2004). AOX can be generated endogenously (e.g., SOD) or come from dietary sources and thus could be regulated in quite different ways (Prior et al. 2007; Cohen et al. 2009). Timing of sampling is also likely to influence the direction of any correlation. This is because the AOX present in plasma can become suppressed by oxidants if sampling occurs too long after the induction of OS (Chung et al. 2013), and different markers might represent acute, rather than chronic oxidative damage (Seet et al. 2011).

Although our study focused entirely on OS markers measured in plasma, there is evidence to suggest that oxidative damage and AOX levels can vary across tissues and among species subjected to the same oxidative insult, or indeed antioxidant supplementation (e.g., Argüelles et al. 2004; Oruc et al. 2004; Catoni et al. 2008; Veskoukis et al. 2009; Yang et al. 2013; Tkachenko et al. 2014; Xu et al. 2014). The potential discrepancies in OS measures among tissues are a serious concern, and ideally, a selection of tissues should be applied to give a better reflection of the overall redox state of an animal. However, in longitudinal studies and studies in the wild, blood is by far the most commonly used tissue to investigate OS as most other tissues require destructive sampling. Turnover of plasma and serum constituents is very high, and thus, measures of OS taken in these tissues may reflect relatively recent redox state, rather than long‐term state (Xu et al. 2014). Correlations among OS markers may be stronger and more consistent if measured in other tissues with slower turnover, but measurements from blood are likely to continue to be the mainstay of OS research within evolutionary ecology. Our findings suggest generalization beyond the specific OS markers measured from blood samples within a study may be unwise.

Although we measured only four out of a very large number of potential OS markers, and only in plasma under highly variable natural conditions, our data still constitute one of the largest and most detailed studies of OS collected from a natural population. Our four markers appear to vary in divergent ways across the 4‐year study period, and we found no evidence for substantive or consistent covariation among the markers within years, age groups, or sexes. Our results emphasize the importance of applying multiple assays of OS as different physiological or environmental conditions may evoke different responses in various components of the redox system. They also caution against generalization beyond the specific markers being measured and suggest much further work is required if we are to understand how the manifold, complex biochemical processes underpinning organism‐wide OS influence life‐history traits and fitness.

## Data Accessibility

Data available from the Dryad Digital Repository: http://dx.doi.org/10.5061/dryad.6782r.

## Conflict of Interest

None declared.

## Supporting information


**Appendix S1.** Intraplate repeatability of Bradford assay.

**Appendix S2.** Interplate repeatability of MDA assay.

**Appendix S3.** Among‐year variation in marker data.
