# Supplementary material for: Plasma markers of oxidative stress are uncorrelated in a wild mammal
Source: Ecol Evol. 2015 Oct 19;5(21):5096–108. doi: 10.1002/ece3.1771 (PMC4662306; doi:10.1002/ece3.1771)
Supplement: Supplementary file 1 — Appendix S1. Intraplate repeatability of Bradford assay. [file ECE3-5-5096-s001.docx]

Intra-plate repeatability (r^2^) of plasma protein content, as measured using the of the non-kit Bradford assay (Bradford, 1976) was calculated (following the methods described by Lessells & Boag, 1987) from 22 samples run in triplicate, taken in August of 2013. **r^2^ = 0.92.**

| Anova single-factor output | | | | | | |
| --- | --- | --- | --- | --- | --- | --- |
| *Source of variation* | SS | df | MS | F | P-value | F crit |
| Between Groups | 0.247 | 21 | **0.012** | 35.723 | 4.94E-21 | 1.801 |
| Within Groups | 0.015 | 44 | **0.0003** |  |  |  |
|  |  |  |  |  |  |  |
| Total | 0.262 | 65 |  |  |  |  |

S^2^ = 0.0003

S^2^A = (Between Groups MS – Within Groups MS)/ 3 = 0.004

**r^2^** = S^2^A/(S^2^+S^2^A) = **0.92**
