# Supplementary material for: Plasma markers of oxidative stress are uncorrelated in a wild mammal
Source: Ecol Evol. 2015 Oct 19;5(21):5096–108. doi: 10.1002/ece3.1771 (PMC4662306; doi:10.1002/ece3.1771)
Supplement: Supplementary file 2 — Appendix S2. Interplate repeatability of MDA assay. [file ECE3-5-5096-s002.docx]

Inter-plate repeatability (r^2^) of malondialdehyde levels of the non-kit HPLC assay was calculated (following the methods described by Lessells & Boag, 1987) from 58 repeat samples, taken in either August 2012 (♦) (n= 34) or 2013 (●) (n= 24). Samples were selected at random and divided into two aliquots, ‘A’ and ‘B’. r^2^ = 0.91.
