# Supplementary material for: Plasma markers of oxidative stress are uncorrelated in a wild mammal
Source: Ecol Evol. 2015 Oct 19;5(21):5096–108. doi: 10.1002/ece3.1771 (PMC4662306; doi:10.1002/ece3.1771)
Supplement: Supplementary file 3 — Appendix S3. Among‐year variation in marker data. [file ECE3-5-5096-s003.docx]

|  | 2010 | | | | 2011 | | | | 2012 | | | | 2013 | | | |
| --- | --- | --- | --- | --- | --- | --- | --- | --- | --- | --- | --- | --- | --- | --- | --- | --- |
| Overall | | | | | | | | | | | | | | | | |
| Marker | **PC** | **MDA** | **TAC** | **SOD** | **PC** | **MDA** | **TAC** | **SOD** | **PC** | **MDA** | **TAC** | **SOD** | **PC** | **MDA** | **TAC** | **SOD** |
| Mean | 0.44 | 1.83 | 1.42 | 4.30 | 0.33 | 1.92 | 2.16 | 10.51 | 1.21 | 1.25 | 2.02 | 6.16 | 0.52 | 1.41 | 1.85 | 7.55 |
| Variance | 0.05 | 0.24 | 0.74 | 3.17 | 0.03 | 0.30 | 1.02 | 14.08 | 0.13 | 0.16 | 0.47 | 4.22 | 0.06 | 0.20 | 2.28 | 12.15 |
| SD | 0.23 | 0.49 | 0.86 | 1.79 | 0.18 | 0.55 | 1.01 | 3.75 | 0.36 | 0.40 | 0.68 | 2.06 | 0.24 | 0.45 | 1.51 | 3.49 |
| Lambs, females and males | | | | | | | | | | | | | | | | |
| Marker | **PC** | **MDA** | **TAC** | **SOD** | **PC** | **MDA** | **TAC** | **SOD** | **PC** | **MDA** | **TAC** | **SOD** | **PC** | **MDA** | **TAC** | **SOD** |
| Mean | 0.37 | 1.81 | 1.55 | 4.32 | 0.35 | 1.86 | 1.64 | 11.53 | 1.07 | 1.27 | 1.63 | 7.71 | 0.55 | 1.46 | 1.82 | 7.96 |
| Variance | 0.03 | 0.32 | 0.63 | 4.23 | 0.04 | 0.27 | 0.27 | 18.21 | 0.10 | 0.09 | 0.19 | 4.64 | 0.07 | 0.21 | 2.65 | 15.73 |
| SD | 0.18 | 0.57 | 0.80 | 2.06 | 0.20 | 0.52 | 0.52 | 4.27 | 0.31 | 0.31 | 0.44 | 2.15 | 0.26 | 0.46 | 1.63 | 3.97 |
| Lambs, females | | | | | | | | | | | | | | | | |
| Marker | **PC** | **MDA** | **TAC** | **SOD** | **PC** | **MDA** | **TAC** | **SOD** | **PC** | **MDA** | **TAC** | **SOD** | **PC** | **MDA** | **TAC** | **SOD** |
| Mean | 0.38 | 1.73 | 1.68 | 4.06 | 0.37 | 1.94 | 1.60 | 11.90 | 1.05 | 1.25 | 1.58 | 7.72 | 0.50 | 1.46 | 1.87 | 8.14 |
| Variance | 0.03 | 0.35 | 0.55 | 4.30 | 0.04 | 0.25 | 0.21 | 17.21 | 0.08 | 0.06 | 0.20 | 4.97 | 0.05 | 0.29 | 3.39 | 18.98 |
| SD | 0.18 | 0.59 | 0.74 | 2.07 | 0.20 | 0.50 | 0.46 | 4.15 | 0.29 | 0.25 | 0.45 | 2.23 | 0.22 | 0.54 | 1.84 | 4.36 |
| Lambs, males | | | | | | | | | | | | | | | | |
| Marker | **PC** | **MDA** | **TAC** | **SOD** | **PC** | **MDA** | **TAC** | **SOD** | **PC** | **MDA** | **TAC** | **SOD** | **PC** | **MDA** | **TAC** | **SOD** |
| Mean | 0.35 | 1.90 | 1.37 | 4.65 | 0.33 | 1.78 | 1.68 | 11.18 | 1.11 | 1.29 | 1.70 | 7.70 | 0.56 | 1.47 | 1.76 | 7.77 |
| Variance | 0.03 | 0.29 | 0.70 | 4.16 | 0.04 | 0.27 | 0.33 | 19.31 | 0.12 | 0.14 | 0.18 | 4.39 | 0.09 | 0.14 | 1.94 | 12.67 |
| SD | 0.18 | 0.54 | 0.84 | 2.04 | 0.19 | 0.52 | 0.57 | 4.39 | 0.34 | 0.38 | 0.42 | 2.09 | 0.30 | 0.37 | 1.39 | 3.56 |
| Adults, females and males | | | | | | | | | | | | | | | | |
| Marker | **PC** | **MDA** | **TAC** | **SOD** | **PC** | **MDA** | **TAC** | **SOD** | **PC** | **MDA** | **TAC** | **SOD** | **PC** | **MDA** | **TAC** | **SOD** |
| Mean | 0.53 | 1.87 | 1.27 | 4.27 | 0.32 | 1.96 | 2.48 | 9.89 | 1.29 | 1.25 | 2.23 | 5.34 | 0.50 | 1.34 | 1.88 | 7.07 |
| Variance | 0.06 | 0.15 | 0.84 | 1.99 | 0.03 | 0.33 | 1.21 | 10.60 | 0.13 | 0.20 | 0.49 | 2.08 | 0.04 | 0.19 | 1.88 | 7.70 |
| SD | 0.25 | 0.39 | 0.92 | 1.41 | 0.16 | 0.57 | 1.10 | 3.26 | 0.36 | 0.44 | 0.70 | 1.44 | 0.20 | 0.43 | 1.37 | 2.78 |
| Adults, females | | | | | | | | | | | | | | | | |
| Marker | **PC** | **MDA** | **TAC** | **SOD** | **PC** | **MDA** | **TAC** | **SOD** | **PC** | **MDA** | **TAC** | **SOD** | **PC** | **MDA** | **TAC** | **SOD** |
| Mean | 0.56 | 1.82 | 1.23 | 4.21 | 0.34 | 1.96 | 2.56 | 10.42 | 1.29 | 1.24 | 2.23 | 5.38 | 0.51 | 1.31 | 1.85 | 7.41 |
| Variance | 0.07 | 0.15 | 0.91 | 2.04 | 0.03 | 0.34 | 1.12 | 11.29 | 0.13 | 0.19 | 0.51 | 2.24 | 0.04 | 0.18 | 1.97 | 7.11 |
| SD | 0.26 | 0.38 | 0.96 | 1.43 | 0.16 | 0.58 | 1.06 | 3.36 | 0.37 | 0.43 | 0.71 | 1.50 | 0.20 | 0.42 | 1.41 | 2.67 |
| Adults, males | | | | | | | | | | | | | | | | |
| Marker | **PC** | **MDA** | **TAC** | **SOD** | **PC** | **MDA** | **TAC** | **SOD** | **PC** | **MDA** | **TAC** | **SOD** | **PC** | **MDA** | **TAC** | **SOD** |
| Mean | 0.38 | 2.19 | 1.50 | 4.70 | 0.27 | 1.95 | 2.25 | 8.44 | 1.27 | 1.25 | 2.23 | 5.14 | 0.47 | 1.52 | 2.00 | 5.26 |
| Variance | 0.03 | 0.08 | 0.34 | 0.82 | 0.02 | 0.30 | 1.43 | 6.01 | 0.10 | 0.26 | 0.42 | 1.40 | 0.04 | 0.21 | 1.50 | 7.41 |
| SD | 0.18 | 0.28 | 0.59 | 0.35 | 0.14 | 0.55 | 1.19 | 2.45 | 0.32 | 0.51 | 0.65 | 1.18 | 0.21 | 0.46 | 1.22 | 2.72 |
